# Seed Priming Boost Adaptation in Pea Plants under Drought Stress

**DOI:** 10.3390/plants10102201

**Published:** 2021-10-17

**Authors:** Sally A. Arafa, Kotb A. Attia, Gniewko Niedbała, Magdalena Piekutowska, Salman Alamery, Khaled Abdelaal, Talal K. Alateeq, Mohamed A. M. Ali, Amr Elkelish, Shreen Y. Attallah

**Affiliations:** 1 Department of Agricultural Botany, Faculty of Agriculture, Mansoura University, Mansoura 35516, Egypt; sally86@mans.edu.eg; 2 Department of Biochemistry, College of Science, King Saud University, P.O. Box 2455, Riyadh 11451, Saudi Arabia; salamery@ksu.edu.sa; 3 Rice Biotechnology Lab, RRTC, Field Crops Research Institute, ARC, Sakha, Kafrelsheikh 33717, Egypt; 4 Department of Biosystems Engineering, Faculty of Environmental and Mechanical Engineering, Poznań University of Life Sciences, Wojska Polskiego 50, 60-627 Poznań, Poland; gniewko.niedbala@up.poznan.pl; 5 Department of Geoecology and Geoinformation, Institute of Biology and Earth Sciences, Pomeranian University in Słupsk, Partyzantów 27, 76-200 Słupsk, Poland; magdalena.piekutowska@apsl.edu.pl; 6 PCRS Excellence Center, Plant Pathology and Biotechnology Lab, Department of Agricultural Botany, Faculty of Agriculture, Kafrelsheikh University, Kafrelsheikh 33516, Egypt; 7 Department of Plant Production, College of Food Science and Agriculture, King Saud University, P.O. Box 2455, Riyadh 11451, Saudi Arabia; talateeq@ksu.edu.sa; 8 Department of Horticulture, College of Agriculture, New Valley University, El-Kharga 72511, Egypt; mohamed.ali@agr.nvu.edu.eg; 9 Botany Department, Faculty of Science, Suez Canal University Ismailia, Ismailia 41522, Egypt; amr.elkelish@science.suez.edu.eg; 10 Department of Vegetables, Faculty of Agriculture, Assiut University, Assiut 71515, Egypt; shereen.awad@agr.aun.edu.eg

**Keywords:** antioxidant, *Bacillus*, carrot extract, drought, enzyme activity, pea, silicon

## Abstract

In the present investigation, we study the effect of *Bacillus thuringiensis* MH161336 (10^6–8^ CFU/cm^3^), silicon (25 mL L^−1^), and carrot extract (75 mL L^−1^) as seed primers, individually or in combination, on morphological, physio-biochemical and yield components of drought-stressed pea plants (Master B) during 2019/2020 and 2020/2021 seasons. Our results indicated that drought causes a remarkable reduction in plant height, leaf area, number of leaves per plant, and number of flowers per plant in stressed pea plants during two seasons. Likewise, number of pods, pod length, seeds weight of 10 dried plants, and dry weight of 100 seeds were decreased significantly in drought-stressed pea plants. Nevertheless, seed priming with the individual treatments or in combination boosted the morphological, physio-biochemical, and yield characters of pea plants. The best results were obtained with the *Bacillus thuringiensis* + carrot extract treatment, which led to a remarkable increase in the number of leaves per plant, leaf area, plant height, and number of flowers per plant in stressed pea plants in both seasons. Moreover, pod length, number of seeds per pod, seeds weight of 10 dried plants, and dry weight of 100 seeds were significantly increased as well. *Bacillus thuringiensis* + carrot extract treatment led to improved biochemical and physiological characters, such as relative water content, chlorophyll a, chlorophyll b, regulated the up-regulation of antioxidant enzymes, increased seed yield, and decreased lipid peroxidation and reactive oxygen species, mainly superoxide and hydrogen peroxide, in drought-stressed pea plants.

## 1. Introduction

*Pisum sativum* L. is one of the most essential vegetable winter crops cultivated in Egypt, and most cultivars are grown for fresh and dry seeds because it contains a high amount of protein, vitamins, carbohydrates, and minerals like iron, phosphorus, and zinc [1]. Increasing the growth and yield of pea plants and other economic plants is considered an important target; there are many environmental factors that affect and harm the growth stages of many plants, such as salinity on strawberry, sweet pepper, rice, cucumber, and faba bean [2,3,4,5,6,7], and drought stress on faba bean, barley, sugar beet, maize and wheat plants [8,9,10,11,12,13,14].

Drought is one of the most serious problems in many countries, particularly in Egypt [8]. Under drought conditions, the growth stages of many plants were adversely affected, and many physiological changes were recorded, such as the decline in stem height, leaf number, leaf area, chlorophyll content, and sugar yield [9,14]. Physio-biochemical parameters such as relative water content, proline content, lipid peroxidation, superoxide, hydrogen peroxide, electrolyte leakage, and enzymes activity, as well as yield, were negatively affected under drought conditions in barley, faba bean, sugar beet, maize, wheat, and rice plants [7,12,15,16,17]. The decrease in cholorophyll A and b concentrations and the photosynthetic rate are among the most important physiological changes under water stress [10]. Under drought, the closed stomatal pores led to the decrease in carbon dioxide (CO_2_) uptake which causes a decrease in the photosynthetic rate because of the reduction in the activities of some special enzymes [18]. Additionally, relative water content (RWC) was significantly reduced in barley and bean plants under drought [10,13]. Reactive oxygen species (ROS) such as hydrogen peroxide (H_2_O_2_) and superoxide (O_2_^•−^), malondialdehyde (MDA) and electrolyte leakage (EL), are the main signals of biotic and abiotic stresses [18,19,20,21].

The increase in EL and MDA may be attributed to the oxidative stress of different organelles, like mitochondria and chloroplasts [22]. In addition, the overproduction of ROS is the key initiator of stress-associated lipid peroxidation and, finally, cell death [23]. Under drought stress, Abdelaal et al. [24] discovered remarkable increases in EL and MDA because of the damage of membranes and cytoplasm in water-stressed barley plants and sugar plants [3]. The accumulation of ROS, especially O_2_^•−^ and H_2_O_2_, increased considerably under drought [3,4,5]. Superoxide and hydrogen peroxide can cause damaging effects to the chloroplast, mitochondria, proteins, and nucleic acids, resulting in cell death [24]. The activity of antioxidant enzymes that scavenge ROS with overproduction of the enzymes led to improve the oxidative stress tolerance under stress [25]. One of the significant enzymes involved in the antioxidant mechanisms is superoxide dismutase (SOD), which converts superoxide to hydrogen peroxide and consequently reduces the damage to proteins and DNA [26]. Additionally, catalase (CAT) is an important enzyme; it contributes with SOD in the degradation of hydrogen peroxide, causing a decrease in the production of the highly reactive radicals [27,28,29,30].

Application of plant growth-promoting bacteria (PGPB) such as *Azotobacter, Bacillus* and *Rhizobium* may be used to improve the yield not only under normal, but also under stress conditions [31,32] by the production of antioxidants, phytohormones and vitamins. Plant growth-promoting bacteria can be used in biofertilization [33,34,35] and as a biocontrol agent [36] to improve the yield production of crops under various conditions. Moreover, silicon can increase the growth and productivity of many plants; it is a significant element and leads to increase the growth and yield characters, mainly under stress factors [31,37]. Additionally, silicon treatment led to enhanced assimilation rate of carbon dioxide, and improved leaf number, chlorophyll concentration, and RWC, as well as yield production [2,8,31]. Carrot root extract is a natural plant extract and is considered a significant stimulant for growth and yield in many plants because it contains high amounts of protein, carbohydrates, and vitamins as bio-regulators, such as vitamin A, B1, B6, C, D, and E [38]. Kasim et al. [39] stated that seed priming with carrot extract was more effective in enhancing the physiological characters of drought-stressed faba bean plants. Moreover, carrot extract led to improve the cell membrane under environmental stress factors. Carotenoids contribute to protecting cells from oxidative stress in golden rice plants [40]. Therefore, the objective of our study was to evaluate the influence of *Bacillus thuringiensis*, silicon, and carrot extract on pea plants (Master B) under drought stress associated with the antioxidant defense system, chlorophyll concentration, RWC, and ROS, as well as seed yield.

## 2. Materials and Methods

### 2.1. Experimental Design and Plant Materials

Two field experiments were prepared in a private farm at Gharbia Governorate, Egypt, during the two winter seasons (2019/2020 and 2020/2021) to assess the influence of *Bacillus thuringiensis* MH161336 (10^6–8^ CFU/cm^3^), silicon (25 mL L^−^^1^) as potassium silicate (K_2_SiO_3_), and carrot extract (75 mL L^−^^1^) as seed primers on morphological, physio-biochemical and yield characteristics of pea plants (*Pisum sativum* L.) (Master B) under drought stress (50% of normal irrigation). The control plants were irrigated with 10 irrigations, while the stressed plants received 5 irrigations. The irrigation scheduling was applied according to the soil water status known as the field capacity (FC). During precultivation at the beginning of each season, soil samples were taken by an Auger T-Handle at depths of 0–20 and 20–40 cm from the soil surface to determine the field capacity, wilting point (WP), and soil available water (SAW) in the laboratory of soil sciences. The biochemical and physiological studies were conducted in the Plant Pathology and Biotechnology Laboratory and EPCRS Excellence Center, Faculty of Agriculture, Kafrelsheikh University. The seeds of pea plants (Master B) were obtained from the Legume Division, Sakha, Agricultural Research Centre; the experiment was laid out in a completely randomized design with five replicates. The seeds were sterilized with sodium hypochlorite 2.5% and 70% ethanol and washed five times with distilled water. Pea seeds were prepared in four groups; the first group were primed by soaking in tap water for 10 h (control), the second group were treated with *Bacillus thuringiensis* MH161336 (10^6–8^ CFU/cm^3^) and kept for 6 h at room temperature, the third were primed by soaking in silicon (25 mL L^−^^1^), and the fourth were primed in carrot extract (75 mL L^−^^1^) for 10 h. The concentration of the *Bacillus*, silicon and carrot extract was adjusted according to previous studies. Carrot root extract was prepared using 200 g fresh carrot roots and blended with 640 mL of tap water; then, the mixture was filtered using filter paper, and increased with tap water to one liter (100% carrot extract) [41]. The sowing of pea seeds was done on two sides of the ridge in hills at 10 cm apart (3 seeds per hill) on the 6th and 7th of September in the two seasons; respectively, seeds were thinned to one plant per hill after germination. During soil preparation, organic fertilizer was added at the rate of 48 m^3^ ha^−^^1,^ and the plants were fertilized with recommended doses consistent with the recommendations of the Ministry of Agriculture. The chemical characters of the experimental soil during two growing seasons were studied before conducting the experiments and presented in Table 1. [42].

### 2.2. Morphological Characters

Pea samples were taken (10 plants per treatment) for morphological studies at 70 days from sowing; plant height (cm), leaf area (cm^2^), number of leaves plant^−^^1,^ and number of flowers plant^−^^1^ were recorded.

### 2.3. Yield Characters

Green pods of the plants were harvested at the suitable maturing stage to determine number of pods plant^−^^1^ and pod length, and the number of seeds pod^−^^1^, seeds weight of 10 dried plants, and dry weight of 100 seeds were measured when the plants were dried at the end of the experiment.

### 2.4. Physiological and Biochemical Characters

#### 2.4.1. Chlorophyll a and b Concentration

Assays of chlorophyll a and b concentrations were done in the fifth fresh pea leaf using acetone 80%. The samples of fresh pea leaves were kept in the solution overnight under dark conditions. The measures were determined at 663, 645, and 470 nm. The concentration of chlorophyll a and chlorophyll b was measured according to Lichtenthaler [43].

#### 2.4.2. Maximum Quantum Efficiency of PS II (Fv/Fm)

Maximum quantum efficiency of PS II (Fv/Fm ratio) was recorded using a chlorophyll fluorometer at 60 days from the sowing. Pea leaves were kept in the dark for 30 min to simulate the reaction of photosystem II. The maximum efficiency of PSII was recorded as the ratio of (Fv) to (Fm) [44].

#### 2.4.3. Determination of Relative Water Content

Ten discs of fresh pea leaves were used to measure RWC. Initially, the fresh weight (FW) for these discs was determined, then the discs were placed in distilled water to determine the turgid weight (TW) after 1 h; the dry weight (DW) was also determined in the dried discs after 24 h at 80 °C. RWC% was measured as follows: RWC = (FW − DW)/(TW − DW) × 100 [45].

#### 2.4.4. Determination of Proline Content

The samples (500 mg) were taken from the fifth fresh pea leaf and placed in 3% sulphosalicylic acid and centrifuged at 3000× *g* for 20 min. Then, 2 mL of glacial acetic acid and 2 mL ninhydrin reagent was boiled for 1 h with 2 mL supernatant. Proline was assayed as μg g^−1^ FW at 520 nm using a spectrophotometer [46].

#### 2.4.5. Determination of Lipid Peroxidation

The samples were taken from the fifth fresh pea leaf (100 mg), placed in 1% trichloroacetic acid and centrifuged for 5 min at 10,000× *g*, then 0.5% thiobarbituric acid was added. The samples were boiled for 30 min at 95 °C and placed on an ice bath, and centrifuged at 5000× *g* for 5 min. The measurements of lipid peroxidation were recorded as malondialdehyde (nmol g^−1^ FW) as follows: (MDA) = [6.45 × (A532 − A600) − (0.56 × A450)] × V − 1 W, where W = weight (g), V = volume (cm^3^) [47].

#### 2.4.6. Assay of Electrolyte Leakage (EL%)

Ten discs of fresh pea leaves were placed in 25 cm^3^ deionized water (10 samples per treatment). The samples were shaken for 20 h, and electrical conductivity was measured (initial) for each vial, then the samples were placed in a water bath for 1 h at 80 °C and shaken at 21 °C for 20 h, and final conductivity was recorded. EL % was calculated as the following formula: initial conductivity/final conductivity × 100 [48].

#### 2.4.7. Assay of Hydrogen Peroxide (H_2_O_2_) and Superoxide (O_2_^•−^)

According to Velikova et al. [49], the samples of fresh pea leaves (250 mg of fresh leaves) were homogenized using 5 mL of 5% TCA (trichloroacetic acid). The samples were centrifuged at 12,000× *g* at 4 °C for 15 min for the homogenates. The supernatant was gathered, added to 10 mM potassium phosphate buffer (pH 7.0) + 1 M KI, then hydrogen peroxide was measured spectrophotometrically at 390 nm as nmol g^−1^ FW. To determine superoxide (O_2_^•−^), the samples of fresh pea leaves (100 mg) were immersed in 10 mM K-phosphate buffer, pH 7.8, 0.05% NBT and 10 mM NaNO_3_ for 1 h at room temperature. The immersed solution (two millilitres) was heated for 15 min at 85 °C and cooled rapidly. Super oxide was measured spectrophotometrically at 580 nm (nmol g^−1^ FW).

#### 2.4.8. Assay of Catalase (CAT) and Superoxide Dismutase (SOD) Activity

From the fifth fresh leaf, 0.5 g was taken, homogenized and centrifuged for 20 min at 12,000× *g*, then the total soluble enzyme activities were measured in the supernatant using a spectrophotometer [50]. CAT activity was assayed in the supernatant at 240 nm as µmol min^−1^ mg protein^−1^ based on the consumption of H_2_O_2_ [51]. The activity of SOD was determined at 560 nm as µmol min^−1^ mg protein^−1^ [52].

### 2.5. Statistical Analysis

The obtained data were analyzed with one-way analysis of variance (ANOVA) procedures [53], using the MSTAT-C statistical software package. The means between treatments were compared with Duncan’s post hoc test when the result of the omnibus multivariate F-test in one-way analysis of variance (ANOVA) was significant (*p* ≤ 0.05) [54].

## 3. Results

### 3.1. Effect of Bacillus thuringiensis, Silicon and Carrot Extract on Plant Height, Leaf Number Plant^−1^, and Leaf Area Plant^−1^ of Drought-Stressed Pea Plants

**Figure 1:**
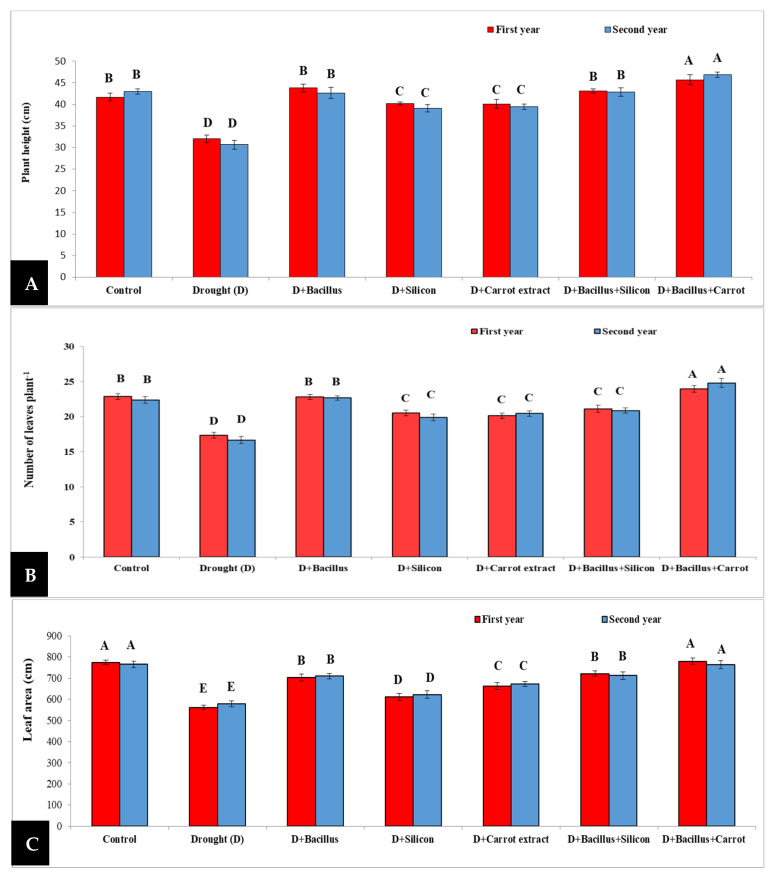
Effect of *Bacillus thuringiensis*, silicon, and carrot extract on plant height (**A**), number of leaves (**B**), and leaf area (**C**) of drought-stressed pea plants during 2019/2020 and 2020/2021 seasons. Different letters on the columns show significant differences between the treatments according to ANOVA, and Duncan’s post hoc test when the result of the omnibus multivariate F-test in ANOVA was significant (*p* ≤ 0.05). Data is the mean (±SE) of five replicates.

Our obtained results revealed that drought led to a remarkable decrease in leaf area, plant height and number of leaves plant^−^^1^ compared to the control treatment (Figure 1); nevertheless, seed priming with *Bacillus thuringiensis*, silicon, and carrot extract, individually or in combination (*Bacillus thuringiensis* + silicon or *Bacillus thuringiensis* + carrot extract), showed a significant increase in the number of leaves plant^−^^1^, plant height (cm) and leaf area plant^−^^1^ compared to the stressed untreated plants during 2019/2020 and 2020/2021 seasons. The highest values of plant height were obtained with *Bacillus thuringiensis* + carrot extract treatment, and the mean of increase during two seasons was 46.5%, followed by *Bacillus thuringiensis* treatment (37.14%), and then *Bacillus thuringiensis* + silicon (36.5%), when compared with stressed untreated plants. Additionally, the combination of *Bacillus thuringiensis* + carrot extract gave the highest value of leaf number with an increase of 44.11%, followed by *Bacillus thuringiensis* (33.7%), then the control treatment (33.11%), as compared with stressed untreated pea plants. Moreover, leaf area was increased significantly in stressed treated plants and the increase was 35.08% in the plants treated with *Bacillus thuringiensis* + carrot extract, compared with stressed untreated plants, followed by *Bacillus thuringiensis* + silicon (25.78%) then *Bacillus thuringiensis* treatment (24%), compared to stressed untreated pea plants during both seasons.

### 3.2. Effect of Bacillus thuringiensis, Silicon and Carrot Extract on Flowers Number Plant^−1^, Pods Number Plant^−1^ and Pod Length of Drought-Stressed Pea Plants

**Figure 2:**
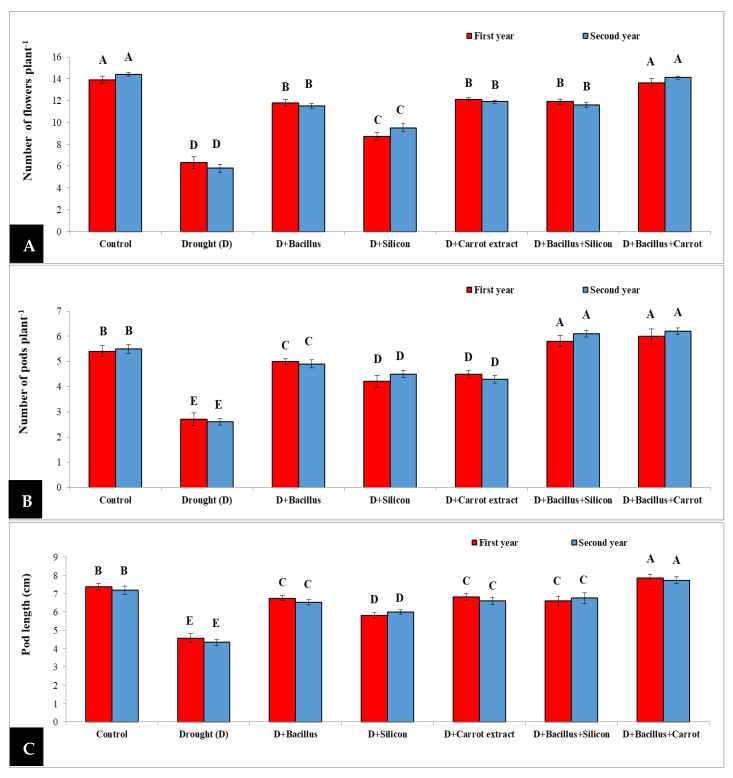
Effect of *Bacillus thuringiensis*, silicon and carrot extract on number of flowers plant^−1^ (**A**), number of pods plant^−1^ (**B**) and pod length (**C**) of drought-stressed pea plants during 2019/2020 and 2020/2021 seasons. Different letters on the columns show significant differences between the treatments according to ANOVA, and Duncan’s post hoc test when the result of the omnibus multivariate F-test in ANOVA was significant (*p* ≤ 0.05). Data is the mean (±SE) of five replicates.

Drought stress led to remarkable decreases in number of flowers plant^−^^1^, number of pods plant^−^^1^ and pod length in stressed pea plants, compared with the control in both seasons (Figure 2). However, seed priming with *Bacillus thuringiensis*, silicon, and carrot extract, individually or in combination (*Bacillus thuringiensis* + silicon or *Bacillus thuringiensis* + carrot extract), led to a significant increase in the number of flowers plant^−^^1^, number of pods plant^−^^1^ and pod length in pea plants under drought. The treatment with *Bacillus thuringiensis* + carrot extract was superior, and the mean of increase during both seasons was 128.9% for the number of flowers plant^−^^1^, 130.18% for the number of pods plant^−^^1^, and 74.88% in pod length, compared with stressed untreated pea plants, followed by the control treatment, then *Bacillus thuringiensis* + silicon and *Bacillus thuringiensis*.

### 3.3. Effect of Bacillus thuringiensis, Silicon and Carrot Extract on Number of Seeds Pod^−1^, Seeds Weight of 10 Dried Plants and Dry Weight of 100 Seeds of Drought-Stressed Pea Plants

**Figure 3:**
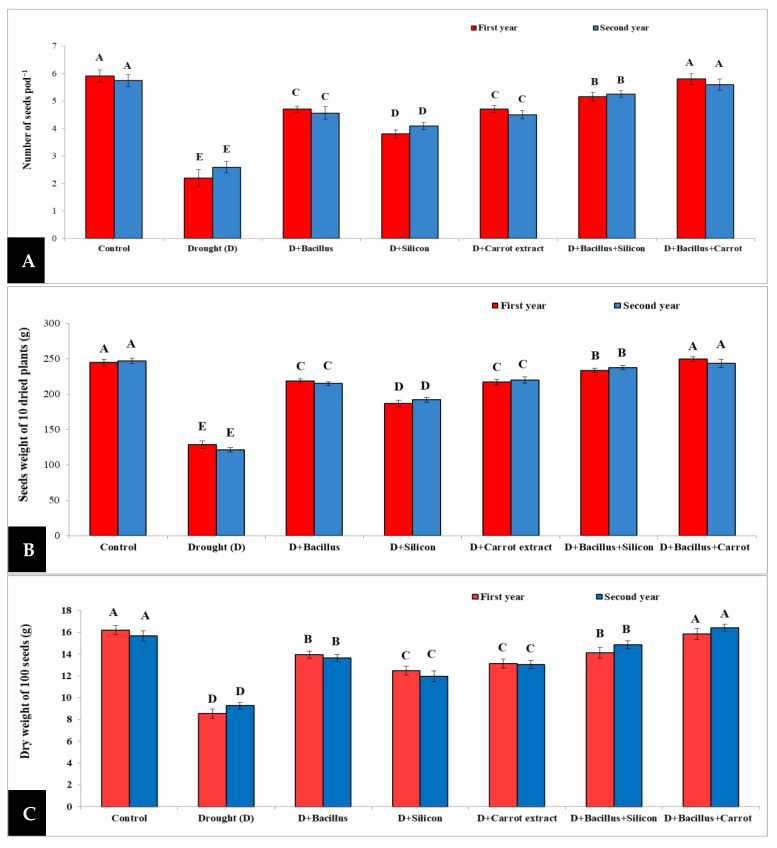
Effect of *Bacillus thuringiensis*, silicon and carrot extract on number of seeds pod^−1^ (**A**), seeds weight of 10 dried plants (**B**), and dry weight of 100 seeds (**C**) of drought-stressed pea plants during 2019/2020 and 2020/2021 seasons. Different letters on the columns show significant differences between the treatments according to ANOVA, and Duncan’s post hoc test when the result of the omnibus multivariate F-test in ANOVA was significant (*p* ≤ 0.05). Data is the mean (±SE) of five replicates.

The presented results in Figure 3 indicated that drought stress caused a significant decrease in number of seeds pod^−^^1^, seeds weight of 10 dried plants and dry weight of 100 seeds of pea plants. However, application of *Bacillus thuringiensis*, silicon and carrot extract, individually or in combination (*Bacillus thuringiensis* + silicon or *Bacillus thuringiensis* + carrot extract), as seed primers led to mitigation of the negative effect of drought on number of seeds, seeds weight of 10 dried plants, and dry weight of 100 seeds of stressed pea plants during two seasons. Regarding our results, seed priming with *Bacillus thuringiensis* + carrot extract gave the maximum values of the number of seeds pod^−^^1^, seeds weight of 10 dried plants and dry weight of 100 seeds of stressed pea plants without any significant difference when compared to control, followed by *Bacillus thuringiensis* + silicon, then the individual treatments in both seasons. The best result of the number of seeds pod^−^^1^ as the mean of both seasons was 130.18%, the seeds weight of 10 dried plants was 95.86%, and the dry weight of 100 seeds was 81.46%, when compared with stressed untreated plants.

### 3.4. Effect of Bacillus thuringiensis, Silicon and Carrot Extract on Chlorophyll a, b Concentration and Maximum Quantum Efficiency of PS II (Fv/Fm) of Drought-Stressed Pea Plants

**Figure 4:**
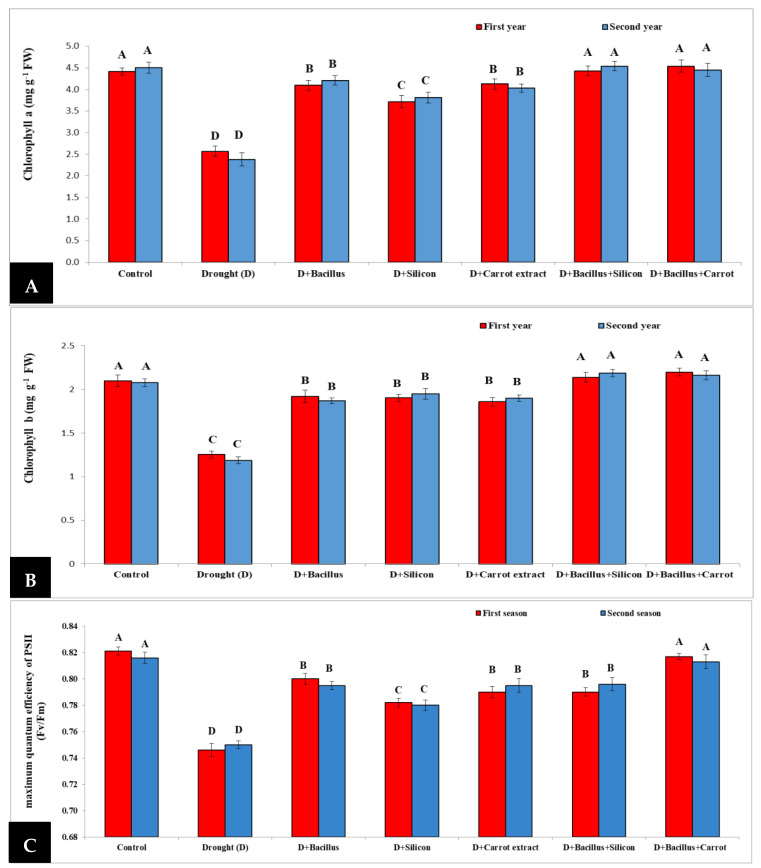
Effect of *Bacillus thuringiensis*, silicon and carrot extract on chlorophyll a (**A**), chlorophyll b (**B**) and maximum quantum efficiency of PS II (**C**) of drought-stressed pea plants during 2019/2020 and 2020/2021 seasons. Different letters on the columns show significant differences between the treatments according to ANOVA, and Duncan’s post hoc test when the result of the omnibus multivariate F-test in ANOVA was significant (*p* ≤ 0.05). Data is the mean (±SE) of five replicates.

Our findings in Figure 4 exhibited that the concentration of chlorophyll a, b and maximum quantum efficiency of PS II (Fv/Fm) considerably decreased in pea plants under drought conditions compared with control plants during two seasons. Conversely, seed priming with all treatments caused a significant increase in chlorophyll a and b, as well as maximum quantum efficiency of PS II. In this regard, the maximum values of chlorophyll a were obtained with *Bacillus thuringiensis* + carrot extract (72%) and *Bacillus thuringiensis* + silicon (71.2%) without any significant difference (71.52%) when compared to the control treatment during the two seasons. Additionally, chlorophyll b was increased significantly because of treatment with *Bacillus thuringiensis* + carrot extract (78.68%) and *Bacillus thuringiensis* + silicon (77.04%), followed by individual treatments in stressed pea plants. Moreover, the high level of maximum quantum efficiency of PS II was observed during both seasons with *Bacillus thuringiensis* + carrot treatment (8.66) and the control, followed by the other treatments, when comparing with stressed untreated plants.

### 3.5. Effect of Bacillus thuringiensis, Silicon and Carrot Extract on Relative Water Content (RWC), Proline Concentration and Lipid Peroxidation (MDA) of Drought-Stressed Pea Plants

**Figure 5:**
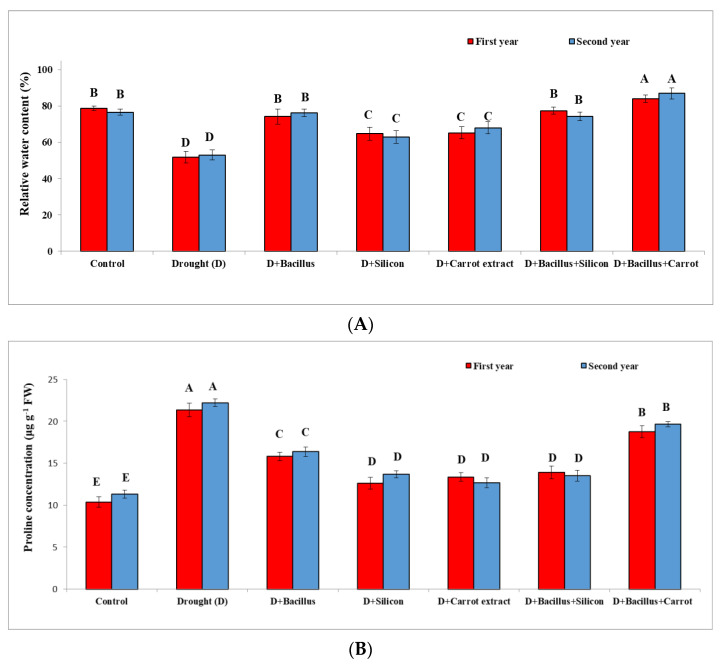

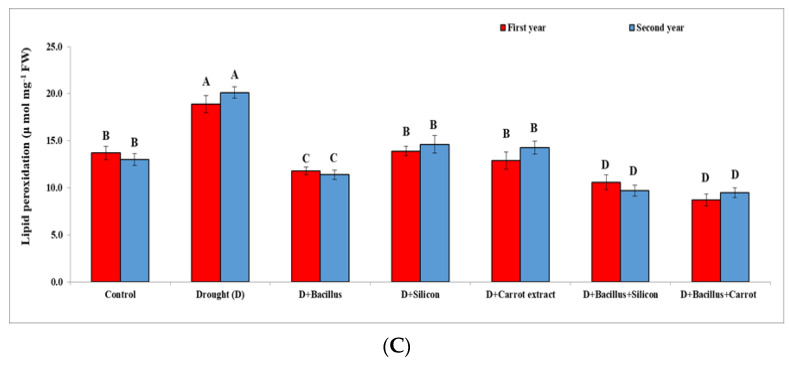
Effect of *Bacillus thuringiensis*, silicon, and carrot extract on relative water content (**A**), proline concentration (**B**) and lipid peroxidation (**C**) of drought-stressed pea plants during 2019/2020 and 2020/2021 seasons. Different letters on the columns show significant differences between the treatments according to ANOVA, and Duncan’s post hoc test when the result of the omnibus multivariate F-test in ANOVA was significant (*p* ≤ 0.05). Data is the mean (±SE) of five replicates.

Drought stress had a significant effect on relative water content (RWC), proline concentration and lipid peroxidation (MDA), as indicated in Figure 5. Relative water content was significantly reduced in pea plants under drought compared to control treatments during two seasons. Conversely, RWC was increased significantly in stressed pea plants due to seed priming with all treatments; the highest level of RWC of the two seasons was achieved with *Bacillus thuringiensis* + carrot extract (63.47%), followed by control plants (48.37%), then the other treatments during both seasons. Regarding proline concentration, the stressed pea plants displayed a remarkable increase in proline during the two seasons. However, seed priming with *Bacillus thuringiensis* + carrot extract and *Bacillus thuringiensis* + silicon and the other treatments led to regulated proline concentration in the stressed pea plants, compared with the stressed untreated plants and control treatment during both seasons. According to various treatments, *Bacillus thuringiensis* + carrot extract decreased proline content (11.98%) compared with stressed untreated plants. MDA was significantly elevated in drought-stressed pea plants during both seasons compared to the control. On the other hand, MDA was decreased significantly with all treatments in the drought-stressed pea plants compared with untreated stressed plants during both seasons. The best treatment was *Bacillus thuringiensis* + carrot extract, which led to a decreased MDA level of 53.58%, and *Bacillus thuringiensis* + silicon (47.94%), which gave the minimum levels of MDA compared with the stressed untreated plants and control treatment.

### 3.6. Effect of Bacillus thuringiensis, Silicon and Carrot Extract on Electrolyte Leakage (EL%), O_2_^•−^ and H_2_O_2_ Level of Drought-Stressed Pea Plants

**Figure 6:**
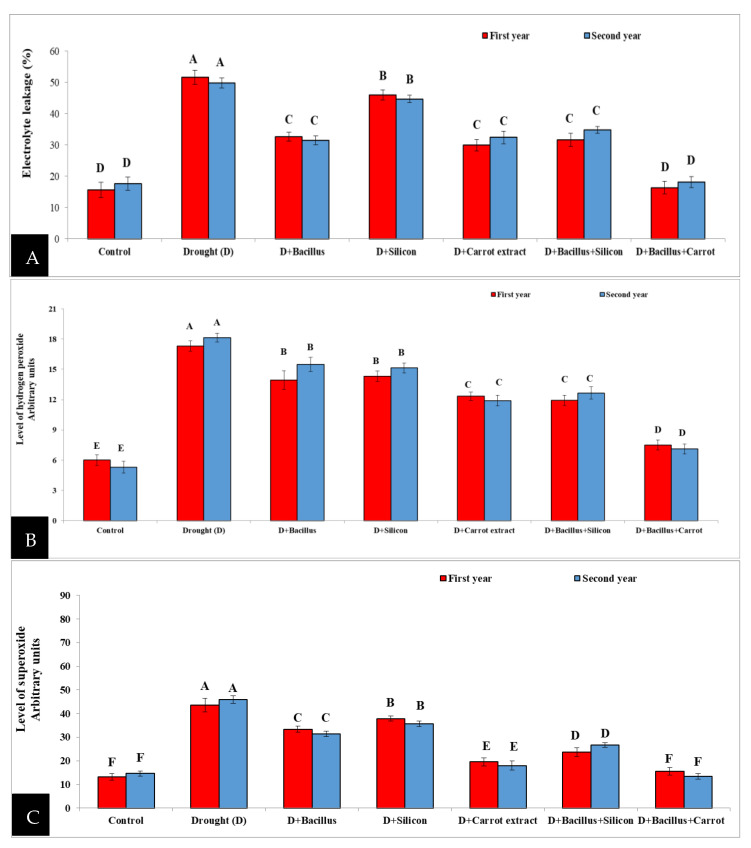
Effect of *Bacillus thuringiensis*, silicon and carrot extract on electrolyte leakage (**A**), hydrogen peroxide level (**B**) and superoxide level (**C**) of drought-stressed pea plants during 2019/2020 and 2020/2021 seasons. Different letters on the columns show significant differences between the treatments according to ANOVA, and Duncan’s post hoc test when the result of the omnibus multivariate F-test in ANOVA was significant (*p* ≤ 0.05). Data is the mean (±SE) of five replicates.

It is obvious from the obtained results in Figure 6 that electrolyte leakage (EL%), O_2_^•−^ and H_2_O_2_ levels were significantly augmented in drought-stressed pea plants. EL% was significantly elevated in pea plants under drought conditions compared with the control during both seasons; however, seed priming led to a significant decrease in electrolyte leakage in drought-stressed pea plants. The best result was achieved with *Bacillus thuringiensis* + carrot extract treatment, and the decrease was (66%) compared with the stressed untreated pea plants, and without any significant difference when compared with the control treatment. Similarly, drought stress caused a significant increase in the levels of O_2_^•−^ and H_2_O_2_ in drought-stressed pea plants compared to the control treatment. Nonetheless, seed priming with different treatments caused a significant decrease in superoxide and hydrogen peroxide levels in drought-stressed pea plants compared to stressed untreated plants. The reduction in hydrogen peroxide according to *Bacillus thuringiensis* + carrot extract treatment was 58.75%, while the decrease in superoxide was 44.55%, as compared to untreated stressed plants as the main of both seasons. Similarly, treatment with *Bacillus thuringiensis* + silicon was in the second level and led to a decrease in hydrogen peroxide of 30.50% and superoxide level of 42.07% in the stressed plants compared with untreated stressed plants.

### 3.7. Effect of Bacillus thuringiensis, Silicon and Carrot Extract on Catalase Activity (CAT) and Superoxide Dismutase (SOD) of Drought-Stressed Pea Plants

**Figure 7:**
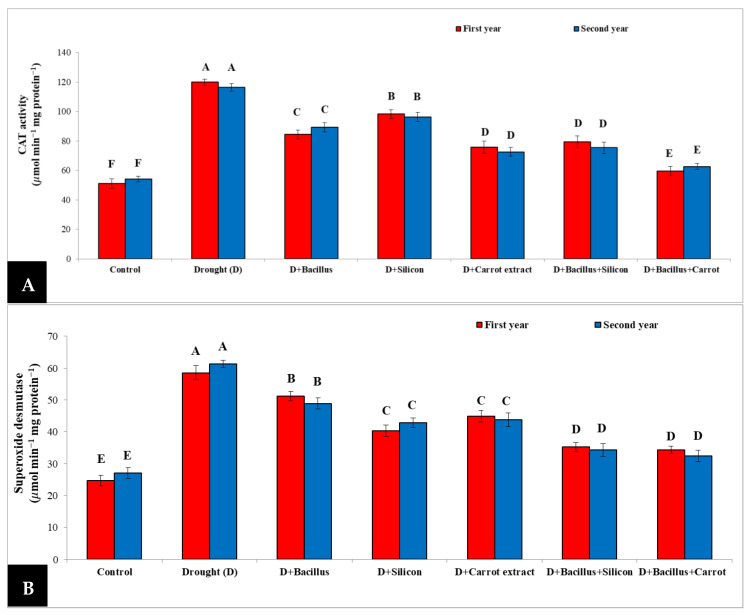
Effect of *Bacillus thuringiensis*, silicon and carrot extract on catalase activity (CAT) (**A**) and superoxide dismutase (SOD) (**B**) of drought-stressed pea plants during 2019/2020 and 2020/2021 seasons. Different letters on the columns show significant differences between the treatments according to ANOVA, and Duncan’s post hoc test when the result of the omnibus multivariate F-test in ANOVA was significant (*p* ≤ 0.05). Data is the mean (±SE) of five replicates.

In the current study, the results in Figure 7 point out that drought stress caused a significant increase in enzyme activity, such as CAT and SOD, in pea plants during both seasons. Catalase activity significantly augmented in drought-stressed pea plants when compared with the control during two seasons. Nevertheless, seed priming with *Bacillus thuringiensis* + carrot extract and other treatments led to a decrease in the activity of CAT in drought-stressed pea plants; the best result was recorded with *Bacillus thuringiensis* + carrot extract, which decreased CAT activity to 47.69% compared with stressed untreated plants. Furthermore, SOD activity considerably augmented in stressed pea plants as compared with the control treatment; however, the activity of SOD was significantly reduced (43.94%) in drought-stressed pea plants due to treatment with *Bacillus thuringiensis* + carrot extract and *Bacillus thuringiensis* + silicon (41.68%), compared to stressed untreated pea plants.

## 4. Discussion

The adverse impacts of drought stress on pea plants were studied in both seasons; drought led to a remarkable decrease in plant height, leaf area, and number of leaves plant^−^^1^. This harmful effect of drought could be attributed to the reduction in water uptake from the rhizosphere and transport through xylem and phloem tissues of pea plants, reduced cell elongation and division, and consequently decreased plant height, number of leaves and leaf area. These results are in line with the results of some researchers in some other plants under drought, such as flax [10], barley [11,15], sugar beet [9], and faba bean plants [8,39]. Additionally, Abdelaal et al. [10] reported that drought stress negatively affected water status in sugar beet plants. Conversely, seed priming with *Bacillus thuringiensis*, silicon, and carrot extract, individually or in combination (*Bacillus thuringiensis* + silicon or *Bacillus thuringiensis* + carrot extract), led to alleviation of the adverse effects of drought on pea plants (Figure 1) and considerably increased plant height, leaf area and number of leaves. The best results were recorded with *Bacillus* + carrot extract treatment in the two seasons; the helpful effect of this treatment may be due to the vital role of *Bacillus* in alleviating the injurious effect of drought and increasing phosphate solubilization and phytohormones [40]. Moreover, carrot extract plays a significant role in producing indole acetic acid, which enhances root structure and increases the concentration of essential elements in stressed pea plants. Furthermore, the role of carrot extract is very significant due to the presence of some essential vitamins such as vitamin A and C, which play a significant role in improving plant status, consequently increasing plant height, leaf area, and number of leaves plant^−^^1^. Similar positive effects of *Bacillus thuringiensis* were observed in lettuce plants under salinity conditions [31], and the positive impacts of carrot extract were recorded in faba bean plants under drought stress [39].

Under drought conditions, the flowering stage and seed yield was considerably affected. All studied parameters such as flower number plant^−^^1^, pod number plant^−^^1^, pod length, seed number pod^−^^1^, seeds weight of 10 dried plants, and dry weight of 100 seeds were significantly reduced in drought-stressed pea plants during both seasons. The reduction in these parameters may be due to the damaging effect of drought on cell division and expansion, leaf and stem size, and the reduction in water and nutrient relations [55,56], which can result in a decrease in flower number plant^−^^1^, pod number plant^−^^1^, pod length, seeds weight of 10 dried plants and the dry weight of 100 seeds. However, a significant increase in these characters was achieved in drought-stressed pea plants with all seed priming treatments (Figure 2 and Figure 3) during two seasons. The best results were achieved with *Bacillus thuringiensis* + carrot extract, followed by *Bacillus thuringiensis* + silicon treatment.

In the present research, our results indicated a significant reduction in chlorophyll a, chlorophyll b concentration, and maximum quantum efficiency of PS II in drought-stressed pea plants during both seasons. This damaging impact of drought may be due to its role in chlorophyll degradation, stomatal closure, and reduced fixation of CO_2_, which causes extreme changes in photosynthesis [57], and consequently decreases chlorophyll a, chlorophyll b concentration, and the maximum quantum efficiency of PS II. The reduction in chlorophyll concentration under drought stress was recorded in various plants, including sugar beet [9], barley [10,24], and wheat plants [16]. Conversely, chlorophyll a and b concentration, and the maximum quantum efficiency of PS II, was considerably elevated in drought-stressed pea plants due to seed priming with *Bacillus thuringiensis* + carrot extract, followed by *Bacillus thuringiensis* + silicon treatment, then silicon and other treatments. The useful effect of *Bacillus thuringiensis* + carrot extract treatment could be attributed to the role of carrot extract in protecting the chlorophyll from oxidative damage under stress due to the presence of carotenoid and vitamin C [40]. Additionally, the positive effect of *Bacillus thuringiensis* could be attributed to its role in improving root growth and water uptake, as well as photosynthesis, under drought stress [40]. Such a positive impact was also recorded by many researchers in several other plants [31,36,40].

Biochemical characters such as proline concentration, electrolyte leakage, superoxide, hydrogen peroxide levels and lipid peroxidation were considerably elevated in pea plants as signals for oxidative stress under drought, while RWC was considerably decreased in pea plants compared with control treatments (Figure 5 and Figure 6). Drought stress had a negative effect on these characters; this effect may be due to the disorder of the plasma membrane and the harmful impact on selective permeability, dehydration of cytoplasm and membrane stability, resulting in decreased relative water content [10]; consequently, this increased the oxidative stress signals, such as proline concentration, MDA, hydrogen peroxide, EL and superoxide level in stressed pea plants in the two seasons. These results agree with the obtained results of some researchers; they found that MDA, proline concentration, EL, O_2_^•−^ and H_2_O_2_ were significantly elevated under stressful conditions in various plants [10,58,59]. Likewise, proline was effective in regulating cell osmotic adjustment and decreasing the levels of ROS under stress [60]. In this regard, Sharma et al. [61] stated that increased proline content lowered ROS under drought stress.

Our results showed that the *Bacillus* with carrot extract treatments lead to more proline accumulation in pea plants. This may due to the *Bacillus* producing many beneficial compounds, for instance plant exopolysaccharides, phytohormones, 1-aminocyclopropane- 1-carboxylate deaminase, and volatile compounds [40,44]. All these compounds trigger the accumulation of all the plants osmolytes (proline), while carrot extract, which serves as a source of antioxidants, has important functions in enhancing plant growth. The β-carotene, which is the main content in carrot extract, which triggers the plants osmolytes, especially proline and glycine betaine [38,39].

Furthermore, antioxidant enzymes such as superoxide dismutase activity (SOD) and catalase (CAT) were considerably increased as a defense mechanism alongside drought stress in pea plants in comparison to the control treatment (Figure 7). The increase in CAT and SOD activity under drought may be due to their involvement in the tolerance of numerous stresses against oxidative damage [62]; they are essential in the scavenging of reactive oxygen species under stress conditions [36]. These findings are in line with the recorded results of Li et al. [63]. In the current study, the detrimental effects of drought can be alleviated by seed priming with *Bacillus*, silicon, and carrot extract, individually or in combination (*Bacillus* + silicon or *Bacillus thuringiensis* + carrot extract). This result could be due to the role of *Bacillus* as a source for cytokinin, auxin and nutrients, as well as the regulation of essential enzymes. Additionally, *Bacillus* can produce exopolysaccharides which improve soil structure, increase water availability [59], and consequently enhance physio-biochemical characters, such as chlorophyll concentration and RWC, and decrease electrolyte leakage superoxide and hydrogen peroxide in pea plants under stress conditions [32,64]. Interestingly, carrot extract can increase α-tocopherol, which may neutralize MDA levels by reducing reactive oxidative anions and increasing membrane stability, and induce and trigger the plant defenses by adjusting the CAT and SOD enzyme activity, which scavenge ROS and protect the cells from oxidative stress. These results are in line with the findings of Kasim et al. [39]; they indicate that carrot extract led to improved faba bean growth, and decreased MDA, CAT and POD activity under drought conditions. Furthermore, there are some important elements in carrot extract such as Ca, Mg, P, and K; it is well known that Mg is necessary for chlorophyll synthesis [65], while Ca can protect the cell membrane under stress conditions [66]. Generally, our findings showed the mitigation of drought effects on pea plants by *Bacillus thuringiensis* + carrot extract treatment; this treatment led to improved growth characteristics and increased yield characteristics of stressed pea plants.

## 5. Conclusions

In general, our results indicated that *Bacillus* MH161336 (10^6–8^ CFU/cm^3^), silicon (25 mL L^−1^), and carrot extract (75 mL L^−1^) as seed primers, individually or in combination, enhanced the morpho-physiological and yield characteristics of pea plants (Master B) by alleviating the adverse impacts of drought stress. Leaf area, plant height (cm), number of leaves plant^−1,^ and number of flowers plant^−1^ were increased significantly in stressed pea plants due to seed priming with the *Bacillus thuringiensis* + carrot extract treatment. Additionally, the number of pods plant^−1^, pod length, number of seeds pod^−1^, seeds weight of 10 dried plants, and dry weight of 100 seeds were significantly increased as well. *Bacillus thuringiensis* + carrot extract treatment led to improved biochemical and physiological characteristics, such as relative water content, chlorophyll a, and chlorophyll b, and regulated the up-regulation of antioxidant enzymes, and decreased lipid peroxidation and reactive oxygen species, mainly hydrogen peroxide and superoxide, in drought-stressed pea plants.

## Figures and Tables

**Table 1:** Chemical characters of the experimental soil during two growing seasons before conducting the experiments.

**Seasons**	**PH**	* EC **Ds/m**	**Mechanical Analysis**			**Organic Matter (%)**	**Total N (%)**	**Total P (ppm)**
			**Sand %**	**Silt %**	**Clay %**			
2019/2020	8.15	0.47	22.13	23.89	47.88	1.79	0.159	0.024
2020/2021	8.04	0.49	22.33	24.12	46.54	1.82	0.151	0.022
Seasons		Soluble cations Meq/L						
		Na^+^	K^+^	Ca^++^	Mg^++^	SO_4_^−−^	Cl^−^	
2019/2020		2.09	0.18	2.07	2.54	2.13	0.46	
2020/2021		2.16	0.15	2.03	2.28	1.86	0.51	

* EC = Electrical conductivity.
